# PCSK9 and the Gut-Liver-Brain Axis: A Novel Therapeutic Target for Immune Regulation in Alcohol Use Disorder

**DOI:** 10.3390/jcm10081758

**Published:** 2021-04-18

**Authors:** Ji Soo Lee, Emma M. O’Connell, Pal Pacher, Falk W. Lohoff

**Affiliations:** 1 Section on Clinical Genomics and Experimental Therapeutics, National Institute on Alcohol Abuse and Alcoholism, National Institutes of Health, Bethesda, MD 20892, USA; jisoo.lee@nih.gov (J.S.L.);; 2 Laboratory of Cardiovascular Physiology and Tissue Injury, National Institute on Alcohol Abuse and Alcoholism, National Institutes of Health, Bethesda, MD 20852, USA; pacher@mail.nih.gov

**Keywords:** AUD, ALD, PCSK9, liver, brain, gut-liver-brain axis, inflammation, neuroinflammation, alcohol-induced neurodegeneration, PCSK9 inhibitor

## Abstract

Alcohol use disorder (AUD) is a chronic relapsing disorder characterized by an impaired ability to control or stop alcohol intake and is associated with organ damage including alcohol-associated liver disease (ALD) and progressive neurodegeneration. The etiology of AUD is complex, but organ injury due to chronic alcohol use can be partially attributed to systemic and local inflammation along the gut-liver-brain axis. Excessive alcohol use can result in translocation of bacterial products into circulation, increased expression of pro-inflammatory cytokines, and activation of immune cells, including macrophages and/or microglia in the liver and brain. One potential mediator of this alcohol-induced inflammation is proprotein convertase subtilisin/kexin type 9 (PCSK9). PCSK9 is primarily known for its regulation of plasma low-density lipoprotein cholesterol but has more recently been shown to influence inflammatory responses in the liver and brain. In rodent and post-mortem brain studies, chronic alcohol use altered methylation of the *PCSK9* gene and increased expression of PCSK9 in the liver and cerebral spinal fluid. Additionally, PCSK9 inhibition in a rat model of ALD attenuated liver inflammation and steatosis. PCSK9 may play an important role in alcohol-induced pathologies along the gut-liver-brain axis and may be a novel therapeutic target for AUD-related liver and brain inflammation.

## 1. Introduction

Excessive alcohol use is a major risk factor for morbidity and mortality, leading to approximately 88,000 deaths in the United States annually. Alcohol abuse costs over 200 billion dollars each year, mainly due to lost work productivity and healthcare expenses [1]. Alcohol use disorder (AUD) is a chronic relapsing condition characterized by an impaired ability to control or stop alcohol intake despite significant detrimental social, occupational, or health consequences [2]. Despite being one of the most prevalent mental health disorders worldwide, AUD remains vastly undertreated because of social stigmas, inadequate systematic screenings in primary health care settings, and limited treatment options [3,4,5].

AUD is related to the dysfunction of several organ systems including alcohol-associated liver disease (ALD) and neurodegeneration in the brain. ALD is the most prevalent type of chronic liver disease globally. It begins with mild and reversible alcoholic fatty liver and, through constant chronic liver injury and inflammation, can progress to alcoholic steatohepatitis (ASH)/alcoholic hepatitis (AH), alcoholic cirrhosis (AC), and fibrosis, which ultimately lead to hepatocellular carcinoma (HCC) and alcoholic hepatic failure [6,7,8,9]. Excessive alcohol consumption can promote chronic liver injury, and AUD comorbid with liver disease is extremely common, with the risk of hepatitis C virus-related decompensated cirrhosis increased by two- to four-fold in the presence of AUD [10].

Alcohol also has a neurotoxic effect on the brain, and chronic use can result in cognitive deficits, numbness and pain in the hands and feet, disordered thinking, dementia, and short-term memory loss [11]. Human neuroimaging and post-mortem studies found reduced frontal cortical and white matter regions and enlarged ventricles in the brains of individuals with AUD compared to controls [12,13,14]. Time course studies of neurodegeneration at various periods during chronic alcohol intoxication indicated that neuronal death increased during intoxication at high blood alcohol concentration (BAC) and progressively decreased during withdrawal [15]. Abstinence improved cognition, metabolism, and brain volume, suggesting alcohol-induced neurodegeneration is somewhat reversible [16].

End-organ damage induced by chronic excessive alcohol use can be partially attributed to persistent inflammation. Acute inflammation acts as an essential protective mechanism against infectious agents and injury by recruiting innate immune cells (neutrophils, monocytes, macrophages, and natural killer (NK) cells) to target tissues to remove damaged cells and promote tissue repair and regeneration. However, when this acute immune response is impaired due to the persistent presence of injured cells and inflammation, chronic inflammation occurs. In addition to innate immune cells, adaptive immune cells (NK, B, and T cells) participate in chronic inflammatory responses. Orchestrating responses of innate and adaptive immune cells and their mediators (cytokines and chemokines) contribute to the pathophysiology of chronic human diseases such as ALD and Alzheimer’s disease [17]. Increased expression of pro-inflammatory genes and high levels of circulating pro-inflammatory cytokines are highly associated with the progression of ALD and neurodegeneration [15,18]. Additionally, astrocyte and microglia activation triggered by systemic inflammation causes neuronal injury in an in vivo model of neurodegeneration through the production of large amounts of pro-inflammatory cytokines [19,20].

Here, we discuss how chronic alcohol exposure affects the development of AUD, ALD, and progressive neurodegeneration and examine the effect of alcohol-induced inflammation along the gut-liver-brain axis. We then describe the role of PCSK9 in the liver and brain and the effect of alcohol on PCSK9. Finally, we propose PCSK9 inhibition as a novel therapeutic target to treat ALD and alcohol-induced neurodegeneration.

## 2. Alcohol and Inflammation

### 2.1. Liver

Over 90 percent of consumed alcohol is broken down in the liver, and alcohol has a heavily negative impact on hepatic function [21,22]. Within the liver, alcohol is metabolized by alcohol dehydrogenase (ADH) to acetaldehyde, which is subsequently converted to acetate by acetaldehyde dehydrogenase (ALDH) [22,23]. These two steps of alcohol metabolism are coupled to the reduction of nicotinamide adenine dinucleotide (NAD) to the free radical NADH, which increases the NADH/NAD+ ratio [24,25]. The generation of free radicals during alcohol metabolism creates an environment for oxidative stress, and excessive alcohol consumption alters the redox state of the liver, which facilitates the pathogenesis of fatty liver (steatosis) [25,26].

Alcohol also modulates the activity of transcription factors involved in lipid metabolism. Sterol regulatory element-binding proteins (SREBPs) are transcription factors that control enzymes involved in cholesterol, fatty acid, and triglyceride synthesis [27]. SREBP-1, an isoform of SREBPs, regulates fatty acid generation by alcohol intake. In rat hepatoma cell lines, ethanol exposure increased SREBP-regulated transcription via enhanced levels of mature SREBP-1 protein, and this effect was likely mediated by acetaldehyde. Moreover, mice fed a low-fat diet with ethanol showed a significant rise in levels of mature SREPB-1 protein, increased expression of hepatic lipogenic genes regulated by SREBP-1, and prominent accumulation of hepatic lipid droplets [28].

Alcohol also inhibits peroxisome-proliferator-activated receptors (PPARs), which are transcription factors that act as lipid sensors in the liver. Among its isoforms, PPAR-α regulates lipid oxidation by inducing expression of fatty acid transport proteins in response to the influx of fatty acids [29,30]. Ethanol exposure to hepatoma cells or primary cultured hepatocytes blocked the transcriptional activity of PPAR-α by weakening its DNA-binding ability [31]. Fatty liver and inflammation were observed in a rat model fed ethanol for four weeks but were markedly reduced by treatment with clofibrate, a PPAR-α-activating ligand [32].

In humans, dyslipidemia is a common feature of ALD, and alcohol alters lipid and lipoprotein levels [33,34]. A study of 59 patients with alcoholic liver cirrhosis found serum triglycerides were a marker of severity of liver damage, with higher triglycerides correlated with worse liver outcomes [35]. Conversely, lipoprotein levels tend to decrease with ALD. In several studies, ALD is strongly correlated with decreased high-density lipoprotein cholesterol (HDL-C) [35]. The relationship between cirrhosis and LDL-C is less clear, with some studies finding poor correlation [36] and other studies finding a reduction in LDL-C that was proportionately associated with severity of liver damage [37,38]. This finding is of interest because alcohol consumption is positively correlated with HDL-C and LDL-C levels [39], but when liver damage becomes apparent, HDL-C levels decrease [40,41]. The decrease in lipoproteins with liver disease may, in part, be explained by diminished liver function due to progressive deterioration of the liver [42].

Bacterial lipopolysaccharides (LPS) are endotoxins that play a pathological role in the development of ALD. They are found in the outer membrane of Gram-negative bacteria and are detected by macrophages and monocytes via Toll-like receptor (TLR)-4, a transmembrane protein in innate immune cells. Hepatocytes and Kupffer cells, the resident macrophages of the liver, eliminate the majority of plasma LPS [43]. Chronic alcohol intake increases gut epithelial membrane permeability, impairment of tight junctions between epithelial cells, and structural changes in the gastrointestinal tract, leading to the translocation of LPS and other bacterial products [44]. Elevated plasma LPS was observed in patients with AC [45] and in ethanol-fed rats [46]. *TLR4*^−/−^ mice significantly attenuated alcohol-induced hepatic steatosis and inflammation [47]. Moreover, chronic alcohol-fed rats treated with antibiotics showed reduced circulating LPS and pro-inflammatory cytokine tumor necrosis factor-alpha (TNF-α) levels and improved liver function and liver damage compared to untreated alcohol-fed rats [48]. Probiotics, antibiotics, and fecal microbial transplantation have also shown promising therapeutic effects in the treatment of ALD in humans [49,50].

Leukocyte infiltration in the liver is one of the most important aspects of ALD pathogenesis [51]. The pro-inflammatory chemokine Interleukin (IL)-8 and cytokine IL-17 play critical roles in inducing neutrophil infiltration in ALD. Circulating IL-8 and IL-17 levels were elevated in AUD patients with liver disease [52,53]. In a rat ALD model, chronic alcohol feeding resulted in differentiation of Kupffer cells to M1-type, a classically activated stage of macrophage, which promotes inflammation, and increased TNF-α expression by Kupffer cells upon LPS treatment [54]. Moreover, chronic alcohol consumption suppresses NK cells responsible for antiviral and antitumor function, which contributes to liver fibrosis and liver tumors in AUD patients [55]. Taken together, these studies show that there is converging evidence that alcohol promotes various inflammatory processes that contribute to ALD.

### 2.2. Brain

Although the etiology of AUD is complex, increasing evidence suggests alcohol-induced inflammation is a driver of progressive neuroinflammation. Alcohol alters immune-related gene expression and immune signaling pathways in the brain. Microarray analyses from human post-mortem brains revealed that genes related to immunity were upregulated in the superior frontal cortex of individuals with AUD compared to controls [56,57], and genes involved in glia and inflammation responses were differentially expressed in alcohol-preferring rats [58,59]. Moreover, exposure to alcohol vapor in rats increased TNF-α, IL-6, and chemokine ligand 2 (CCL2) in reward-associated brain areas in sex- and time-dependent manners [60].

Ethanol activates the TLR4 signaling pathway in astrocytes and microglia, which stimulates nuclear factor kappa-light-chain-enhancer of activated B cells (NF-κB), the transcription factor that mediates pro-inflammatory gene expression including IL-6, IL-1β, and TNF-α, and inflammasome regulation to induce neuroinflammation and neurodegeneration and the subsequent production of the cytokines [61]. In astrocyte cultures, the blockade of the TLR4 signaling cascade using neutralizing antibodies reduced the ethanol-induced pro-inflammatory molecules and prevented cell death [62]. In mice, inhibition of TLR4 signaling by the opioid antagonist nalmefene decreased ethanol-induced pro-inflammatory cytokines, chemokines, and mediators in the prefrontal cortex (PFC), striatum, and nucleus accumbens, ultimately abolishing alcohol preference and increased alcohol consumption [63]. Not surprisingly, modulation of the gut microbiota appears to lessen the pro-inflammatory effects of alcohol in the brain by attenuating the translocation of pro-inflammatory bacterial products into systemic circulation and alleviates alcohol-induced anxiety/depression and/or dependence [64,65].

Phosphodiesterase (PDE) and PPAR are also involved in neuroimmune pathways and alcohol consumption. PDE plays an integral role in the regulation of several intracellular signaling pathways by reducing levels of second messengers cyclic adenosine monophosphate (cAMP) and cyclic guanosine monophosphate (cGMP), which may promote excessive alcohol consumption [66]. PDE-4 regulates neuroinflammation triggered by ethanol. Chronic alcohol-fed mice showed markedly increased expression of PDE-4 subfamily B (PDE4B) and decreased cAMP levels in brain tissue, along with vigorous activation of microglia and astrocytes and elevated levels of inflammatory cytokines. Genetic deletion and pharmacological inhibition of PDE4B inhibited ethanol-induced neuroinflammation [67]. PPAR-α is expressed throughout most peripheral tissues as well as in brain regions implicated in AUD [68]. The PPAR-α agonists tesaglitazar and fenofibrate lowered ethanol intake and changed the expression of genes associated with ethanol consumption in the PFC and amygdala of mice [69]. Similar to what is observed in the liver, excessive alcohol use induces neuroinflammation and contributes to central nervous system (CNS) damage.

### 2.3. Gut-Liver-Brain Axis

Alcohol-induced changes in the gut and liver exacerbate the effect of alcohol in the brain and establish a gut-liver-brain axis of inflammation. Systemically, heavy alcohol consumption increases gut permeability, translocation of bacterial products into the splanchnic, and systemic circulation. These changes lead to increased hepatic TNF-α and other pro-inflammatory mediators, which are then transported across the blood-brain barrier (BBB) to activate NF-κB in glial cells [70]. This mechanism is similar to the development of hepatic encephalopathy in chronic liver diseases [71]. Alcohol-induced activation of glia cells and subsequent production of pro-inflammatory mediators can induce neuronal damage [72,73].

The impaired intestinal barrier integrity by alcohol-induced leaky gut leads to intestinal dysbiosis with decreased levels of anti-inflammatory bacteria and increased abundance of proteobacteria, which can affect hepatic and cognitive function [74,75]. In preclinical studies, improved intestinal barrier integrity alleviated alcohol-induced liver damage by reducing intestinal and hepatic oxidative stress and inflammation [76,77]. Moreover, modification of the intestinal environment using prebiotics or antibiotics changed the expression of neurochemicals in the brains of rodents, such as brain-derived neurotrophic factor (BDNF), which plays critical roles in cognitive function [78,79].

One systemic LPS injection resulted in a rapid increase in TNF-α in the liver and brain, increased activated microglia and pro-inflammatory factors in the brain, and degeneration of dopaminergic neurons in the substantia nigra [80]. In the brain, ethanol metabolism by cytochrome P450 2E1 (CYP2E1) increases reactive oxygen species (ROS) levels, which directly activates NF-κB [81]. Prolonged production of ROS and pro-inflammatory cytokines trigger neuronal apoptosis and result in alcohol-induced neurodegeneration [82]. Cognitive impairment due to alcohol-induced cell death in regions such as the PFC is associated with liver dysfunction. Patients with nonalcoholic steatohepatitis have significantly increased lifetime rates of major depressive disorder and generalized anxiety disorder [83]. Additionally, patients with nonalcoholic fatty liver disease have a four times higher risk of cognitive impairment as assessed by the Montreal Cognitive Assessment test (MoCA). Lower MoCA score is also correlated with white and gray matter reduction [84].

In summary, alcohol promotes inflammation in both the brain and liver through alteration of intestinal microbiota, changes in gene expression, and activation of signaling pathways in the innate and adaptive immune system. It creates an axis where gut, liver, and brain inflammation exacerbate each other, resulting in ALD and progressive neurodegeneration.

## 3. PCSK9, Alcohol, and Inflammation

### 3.1. Liver

The most prominent role of PCSK9 is its regulation of plasma low-density lipoprotein cholesterol (LDL-C) homeostasis by targeting the LDL receptor (LDLR) for degradation. PCSK9 circulates in the bloodstream, and when LDLs including LDL-C bind to LDLRs on the surface of hepatocytes, PCSK9 interacts with the epidermal growth factor-like repeat A (EGF-A) domain of the LDLRs. Upon endocytosis, PCSK9 prevents the open extended conformation of LDLR associated with receptor recycling. Instead, the PCSK9/LDLR complex is shuttled to the lysosome for degradation, reducing the concentration of LDLRs on the membrane and subsequently elevating circulating LDL-C levels [85]. Transgenic mice overexpressing PCSK9 in the liver showed elevated plasma cholesterol compared to wild-type mice [86,87]. In line with preclinical findings, genetic studies have reported that gain-of-function mutations in the *PCSK9* gene cause autosomal dominant hypercholesterolemia [88], while loss-of-function mutations in the *PCSK9* gene are closely associated with low LDL-C levels and reduced risk of coronary heart disease (CHD) [89,90].

PCSK9 is expressed not only in the liver and but also in the small intestine along the intestinal cephalocaudal axis [91,92]. Zaid and colleagues found that plasma cholesterol levels were reduced by 42% in total *PCSK9* knockout mice and by 27% in hepatocyte-specific *PCSK9* knockout mice compared to wild-type mice, suggesting that hepatic PCSK9 mainly contributes to cholesterol metabolism [93]. Moreover, a recent study found intestine-specific PCSK9-deficient mice did not show altered postprandial lipemia (PPL) while pharmacological inhibition of circulating PCSK9 in wild-type mice reduced PPL, suggesting that intestine-derived PCSK9 is not a critical regulator of PPL [94].

PCSK9 is also involved in hepatic inflammation. Intraperitoneal injections of LPS in mice increased PCSK9 expression, resulting in decreased LDLR protein in the liver [95]. Reciprocally, exposure of human recombinant PCSK9 in cultured human macrophages and co-culture of the macrophages with hepatic cells overexpressing PCSK9 induced TNF-α and IL-1β expression through LDLRs [96]. These studies imply that PCSK9 and hepatic inflammation may create a feed-forward loop, which exacerbates ALD pathogenesis. NOD-, LRR-, and pyrin domain-containing protein 3 (NLRP3) inflammasome and TLR4/ NFκB signaling pathway participate in PCSK9-mediated inflammation. NLRP3 inflammasome, a critical mediator of host immune response via activation of caspase-1, induced PCSK9 expression through IL-1β in macrophages. The IL-1β-induced PCSK9 secretion involved mitogen-activated protein kinases (MAPKs), extracellular signal-regulated kinase (ERK), Jun kinase (JNK), and p38 [97]. Interference of *PCSK9* gene using PCSK9 shRNA attenuated proinflammatory gene (TNF-α, IL-1β, monocyte chemoattractant protein-1(MCP-1)), TLR4, and NFκB expression and nuclear localization of NFκB in apolipoprotein E (ApoE)-knockout mice [98].

PCSK9 expression was also heightened in patients with liver fibrosis and in a fibrosis mouse model (BDL mice) compared to control groups. In the BDL mice, genetic deletion of PCSK9 via tail vein injection of CRISPR-PCSK9 adeno-associated virus improved liver inflammation and fibrosis with reduced LPS and hepatocyte necrosis markers alanine transaminase (ALT) and aspartate transaminase (AST), suggesting that PCSK9 inhibition can rescue hepatic inflammation and hepatocyte injury [99]. Of interest, in a cohort of human patients with liver cirrhosis secondary to alcohol consumption, serum PCSK9 was reduced compared to non-cirrhotic patients and was not correlated with the severity of liver disease, bilirubin, or aminotransferases, suggesting dynamic expression of PCSK9 throughout liver disease progression [100]. PCSK9 inhibition by alirocumab, a monoclonal antibody against PCSK9, upregulated hepatic LDLR expression and attenuated liver neutrophil and macrophage infiltration, hepatocellular injury, steatosis, and fibrosis in a mouse model of non-alcoholic steatohepatitis [101]. Alirocumab treatment also increased the expression of the VLDL-related gene microsomal triglyceride transfer protein (Mttp), but not several β-oxidation–related genes, suggesting that induction of LDLR following alirocumab treatment may contribute to limiting liver injury by improving VLDL synthesis [101]. In human genetic studies, the PCSK9 rs11591147 loss-of-function (LOF) variant was protective against liver steatosis, nonalcoholic steatohepatitis, and fibrosis [102].

PCSK9 was identified as a primary target epigenetically regulated by alcohol intake in an epigenome-wide association study in individuals with AUD. It was shown that methylomic variations in the promoter region of *PCSK9*, where SREBP-2 and hepatocyte nuclear factor-1α (HNF1α) bind, were associated with expression changes. Mild alcohol exposure was correlated with lower PCSK9 expression whereas chronic alcohol exposure resulted in higher PCSK9 expression [103]. Given the impact of chronic alcohol intake on hepatic lipid metabolism and inflammation, PCSK9 might represent a novel target in the pathophysiology of ALD. In an excessive alcohol-fed rat model, long-term alcohol exposure led to increased hepatic PCSK9 expression, triglyceride (TG), and total cholesterol via activation of SREBP-2 and suppression of extracellular signal-regulated kinase (ERK)1/2 [104]. Moreover, in vitro data suggested that HNF1α was a key modulator of PCSK9 expression and circulating LDL-C levels [105,106].

### 3.2. Brain

Like the liver, PCSK9 plays a role in LDLR metabolism and inflammation in the brain. PCSK9 helps regulate structural and functional development of the brain and is highly expressed in proneural domains (three-to-six somite stage, 10.33–12 h post-fertilization) in zebrafish epiblasts and in the telencephalon (E12.5) and cerebellum (E17-P15) in mouse embryos. In adulthood, PCSK9 is only expressed in areas of continued neurogenesis like cortical, intracranial, and cerebellar granule neurons in zebrafish and the rostral extension of the olfactory peduncle (RE-OP) in mice [92,107,108]. Silencing of *PCSK9* in mouse embryos led to significantly higher levels of LDLR protein in the telencephalon and cerebellum compared to wild-type mice, and levels of untruncated apoE, the principal cholesterol carrier in the brain, were ~25% lower [108]. The effect of PCSK9 in the adult brain is less clear, as silencing and overexpression of PCSK9 in adult mice did not affect LDLR or apoE protein levels in the RE-OP, olfactory bulb, hippocampus, or cortex despite colocalization of *PCSK9* and *LDLR* mRNA [108,109].

PCSK9 is also involved in neuroinflammation. PCSK9 inhibition with the PCSK9 inhibitor (PCSK9i) Prep2-8 trifluoroacetate salt significantly reduced levels of Phospho-NFκB/ NFκB and reactive microglia and astrocytic proliferation and hypertrophy in a rat model of cardiac ischemia/reperfusion injury [110]. Of interest, the PCSK9i did not reduce PCSK9 levels in the brain, suggesting that it did not cross the BBB and modulated neuroinflammation by lowering serum PCSK9 concentrations rather than acting directly in the brain. Another study found that apoE and apoE mimetic interactions with LDLRs reduced LPS-mediated TNF-α and IL-6 secretions in BV2 microglia and human THP-1 monocytes, suggesting that PCSK9 may indirectly mediate local inflammation by controlling LDLR and apoE levels [111]. Furthermore, neuroinflammation is closely connected with depression [112,113,114]. In obese adults, plasma PCSK9 levels were positively associated with depressive symptoms assessed by Beck Depression Inventory (BDI-II) [115].

A study of PCSK9 levels in the cerebrospinal fluid (CSF) of individuals with AUD showed that PCSK9 was significantly increased at days 5 and 21 after admission to an inpatient rehabilitation program compared to controls [116]. In a healthy state, average CSF PCSK9 concentration remains constant over 24 h, while serum PCSK9 concentrations are diurnal and peak in the early morning and afternoon. Of interest, plasma PCSK9 levels were positively correlated with CSF PCSK9 levels in individuals with AUD, while there was no significant correlation between serum and CSF PCSK9 levels in healthy volunteers [116,117]. Hypermethylation of the *PCSK9* gene in response to chronic alcohol consumption, and the resulting elevated expression of PCSK9 in the liver and CNS suggests that PCSK9 plays an important role in AUD pathogenesis.

### 3.3. Gut-Liver-Brain Axis

Chronic alcohol intake impairs insulin signaling in the liver and brain, leading to insulin resistance [118]. Dysbiosis of the gut microbiota can induce PCSK9 expression in the intestine through the LPS-TLR signaling cascade, which can be augmented by insulin resistance [119]. In an epigenome-wide association study of individuals with AUD, the CpG site cg01444643, located in the promoter of the *PCSK9* gene, was identified as a primary target epigenetically regulated by alcohol intake. Additionally, a cross-tissue analysis of *PCSK9* DNA methylation in a mouse model found that there was a significant correlation between brain and liver, but not between blood and brain or blood and liver [103].

PCSK9 is involved in hyperlipidemia-induced neuronal apoptosis. In apoE-deficient mice receiving a high-fat diet (HFD), PCSK9 expression was increased, along with elevated levels of plasma lipids, lipid accumulation, and neuronal apoptosis in the hippocampus [120]. Furthermore, mice fed with HFD showed upregulated serum lipid levels and PCSK9 expression in both the liver and brain. Upon middle cerebral artery occlusion to mimic ischemic stroke, the hyperlipidemic mice exhibited enhanced neuronal apoptosis, cerebral injury, PCSK9, and ApoER2 levels in the brain, which was abrogated by inhibition of PCSK9 using short-hairpin RNA targeting PCSK9 to the cerebral cortex [121]. Hyperlipidemia is highly associated with inflammatory processes, making inflammation a critical modulator in the development of atherosclerotic cardiovascular diseases (ASCVDs). For example, rabbits fed with HFD showed significantly increased serum LDL-C and TNF-α and their positive correlation [122]. The lack of TNF-α in mast cells prevented atherogenesis in *LDLR*^−/−^ mice [123]. Therefore, PCSK9 may link inflammation induced by abnormal lipidemia to neuronal injury and apoptosis.

## 4. PCSK9 Inhibitors as a Potential Therapeutic Target for AUD and ALD

Given the observed increase in PCSK9 with chronic alcohol consumption and the role of PCSK9 in cholesterol regulation and inflammation, inhibition of PCSK9 may be a novel therapeutic approach for AUD and ALD. There are two current PCSK9 inhibitors that are FDA-approved, evolocumab and alirocumab, as well as others that are in phase 3 clinical trials (Table 1). Evolocumab and alirocumab are humanized monoclonal antibodies used for the treatment of adults with heterozygous familial hypercholesterolemia or clinical ASCVD who require additional lowering of LDL-C [124,125]. They interact with circulating free PCSK9 to prevent it from binding to LDLRs, thus lowering plasma LDL cholesterol levels by 50–60%. In patients with a history of ASCVD and statin therapy, PCSK9 inhibitors significantly lowered plasma LDL-C levels and the risk of cardiovascular (CV) events (e.g., CV death, myocardial infarction, stroke) compared to the control group with only the standard therapy [126,127,128,129]. However, meta-analysis and secondary analysis of randomized controlled trials reported that the PCSK9 monoclonal antibodies did not have a significant effect on reduction of high sensitivity C-reactive protein (hs-CRP), an inflammatory biomarker [130].

Although evolocumab and alirocumab do not cross the BBB, there was a concern about these antibodies interfering with cognition due to the importance of cholesterol in brain function [131]. Early phase 2 safety studies, The Open-Label Study of Long-term Evaluation Against LDL-C (OSLER) and The Long-term Safety and Tolerability of Alirocumab in High Cardiovascular Risk Patients with Hypercholesterolemia Not Adequately Controlled with Their Lipid Modifying Therapy (ODYSSEY LONG TERM), reported some neurocognitive impairment with the antibodies, although differences between treatment groups were not statistically significant. Phase 3 clinical trials with larger sample sizes and longer follow-up periods reported there were not significant neurocognitive adverse events associated with PCSK9 inhibitors. The Evaluating PCSK9 Binding antiBody Influence oN coGnitive HeAlth in High cardiovascUlar Risk Subjects (EBBINGHAUS) trial followed 1204 patients over 26 months and found memory or concentration difficulty in 1.9% of the evolocumab group and 1.6% of the placebo group. This difference was not significant, and there was no association between PCSK9i or low LDL-C and neurocognitive decline [128,132]). Additionally, a recent meta-analysis of 14 randomized trials found no change in neurocognition with PCSK9i [133].

To test the possibility of PCSK9 inhibition as a therapeutic option for ALD, one study administered alirocumab to a rat model receiving a chronic alcohol liquid diet. Alirocumab treatment decreased alcohol-induced PCSK9 mRNA expression and upregulated LDL-R through modulation of the transcription factors (e.g., SREBP-1, SREBP-2) in the liver. PCSK9 inhibition with alirocumab attenuated alcohol-induced hepatic TG accumulation through modulation of lipid metabolism, which decreased and increased transcriptional levels of fatty acid synthesis (FAS) and PPARα, respectively. Alirocumab administration also improved ALT and AST levels, ameliorated hepatic inflammation by reducing pro-inflammatory cytokines (e.g., TNF-α and IL-1β), and prevented neutrophil infiltration in chronic alcohol-fed rats [134]. Of note, this monoclonal antibody was given via subcutaneous injection, which would benefit patients with AUD and ALD because there is no metabolism through the liver [126]. PCSK9 may be a promising therapeutic option for AUD and ALD by targeting inflammation caused by alcohol use in multiple organs along the liver–brain axis.

## 5. Discussion

Chronic alcohol use promotes ALD and neurodegeneration through an inflammation feedback loop along the gut-liver-brain axis. Alcohol consumption disrupts the intestinal barrier integrity, which allows the passage of LPS into systemic circulation and subsequently contributes to hepatic and neuroinflammation. Alcohol metabolism in the liver generates free radicals that promote oxidative stress and activate inflammatory genes. In human and rat models, chronic alcohol use increases pro-inflammatory cytokine levels including TNF-α, IL-8, and IL-17 and induces macrophage activation, which further promotes gene expression of pro-inflammatory cytokines [52,54]. In the brain, alcohol increases pro-inflammatory cytokine levels directly and by transport of hepatic TNF-α across the BBB [70].

**Figure 1:**
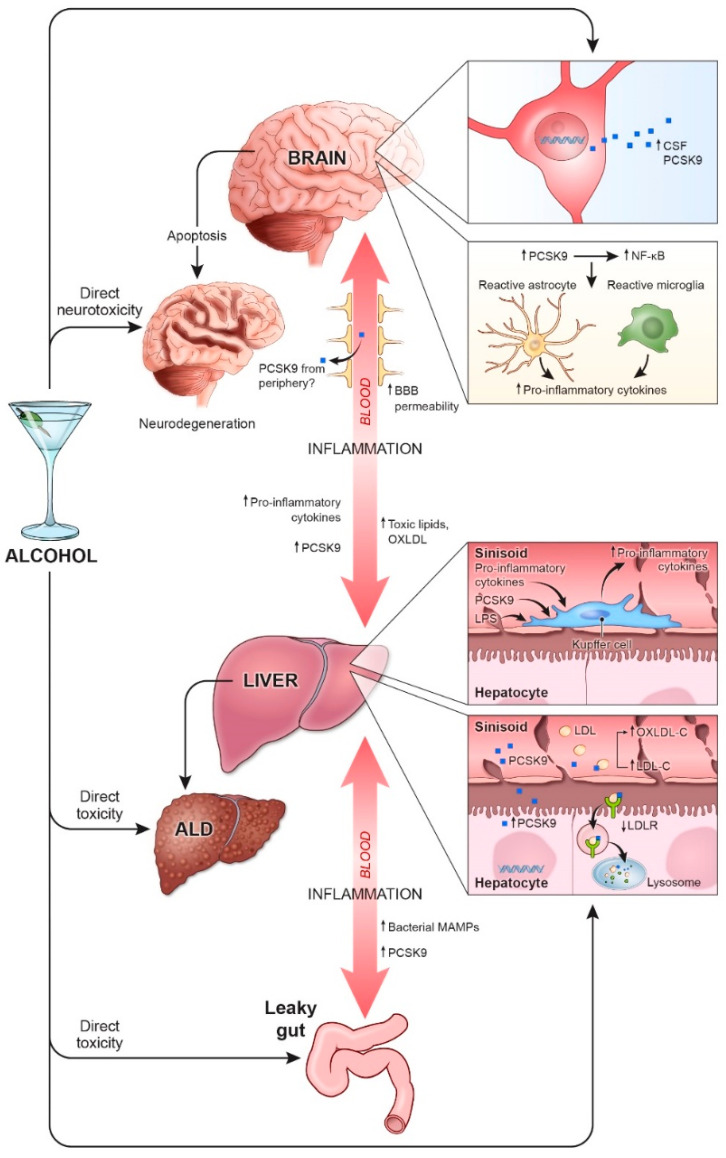
The central role of proprotein convertase subtilisin/kexin type 9 (PCSK9) along the gut-liver-brain axis in alcohol-associated diseases. Alcohol induces brain and liver toxicity and cell death directly and through PCSK9-mediated inflammation. Alcohol is first absorbed in the gut and can disrupt the intestinal barrier integrity, leading to the passage of bacterial microbe-associated molecular patterns (MAMPs) derived from enteric bacterial microbiota into circulation. Dysbiosis in the gut also increases plasma PCSK9 levels. In the liver, alcohol increases PCSK9 protein levels, which leads to increased degradation of low-density lipoprotein receptors (LDLRs) and increased plasma low-density lipoprotein cholesterol (LDL-C) and oxidized low-density lipoprotein cholesterol (oxLDL-C). In addition to alcohol-induced lipopolysaccharides (LPS) and pro-inflammatory cytokines, secreted PCSK9 stimulates Kupffer cells in the liver, which further releases pro-inflammatory cytokines. Elevated levels of PCSK9, pro-inflammatory cytokines, and toxic lipids in the blood promote systemic and neuroinflammation. Plasma PCSK9 leads to increased levels of phosphorylated nuclear factor kappa B (NFκB) and reactive astrocytes and microglia. These glia release more pro-inflammatory cytokines to further perpetuate this axis of inflammation. Additionally, alcohol directly increases PCSK9 levels in the cerebrospinal fluid (CSF). Note: BBB—blood-brain barrier, oxLDL—oxidized low-density lipoprotein, ALD—alcohol-associated liver disease.

The mechanisms underlying alcohol-induced inflammation are complex, but PCSK9 may be involved in perpetuation of ALD and neurodegeneration with alcohol use. PCSK9 is primarily involved in cholesterol regulation in the liver and developing brain [85,108] but more recently has been shown to increase inflammation in the liver and brain [96,110]. Alcohol alters DNA methylation of PCSK9 and increases PCSK9 expression in the gut, liver, and CSF (Figure 1) [103,116]. While it is clear that PCSK9 is involved in mediating the effect of alcohol on the gut-liver-brain axis, the interaction between alcohol and PCSK9 in the gut has not been well studied. It would be valuable for future studies to explore changes in PCSK9 expression and inflammation in the intestinal epithelium with alcohol, given that alcohol is first absorbed in the gut and given the connection between the gut and brain through the enteric nervous system.

Due to its increased expression with chronic alcohol use and its role in inflammation, PCSK9 may be a novel therapeutic target for alcohol-associated diseases. Animal studies have found that alirocumab treatment rescues liver phenotypes in an alcohol-fed rat model and peripheral PCSK9 inhibition prevents astrocyte and microglial activation and neuroinflammation in a rat model of stroke [110,134]. Given that chronic alcohol use makes the BBB more permeable, it is unclear whether the currently available PCSK9 monoclonal antibodies cross the BBB. It would be interesting to explore whether a decrease in neuroinflammation with PCSK9 inhibition is due to a decrease in systemic inflammation or if the inhibitor is working directly in the brain.

More recent PCSK9 inhibitors such as inclisiran are targeted specifically to the liver. Inclisiran is a synthetic small interfering RNA (siRNA) that lowers LDL-C by degrading PCSK9 mRNA using the body’s natural RNA interference pathway. The siRNA is conjugated to triantennary N-acetylgalactosamine carbohydrates that bind to liver-expressed asialoglycoprotein receptors to target inclisiran to hepatocytes [135]. Phase 2 and 3 clinical trials show that inclisiran is just as effective as monoclonal antibodies at lowering cholesterol, reducing LDL-C by about 50%, and requires only one subcutaneous injection every 6 months rather than every two weeks [136,137]. The same siRNA technology could be conjugated to molecules that bind receptors in gut and brain cells to explore the role of PCSK9 on inflammation and lipid dysregulation in different organs and under different conditions. A better understanding of the impact of alcohol on PCSK9 expression and ALD and neurodegeneration may facilitate the clinical development of PCSK9 inhibitors for the treatment of AUD/ALD.

## Figures and Tables

**Table 1:** Ongoing phase 3 clinical trials of PCSK9 inhibitors.

Drug Name	Mechanism of Action	Clinical Trial Identifier	Study Population	Primary Objectives	Primary Completion Date
Alirocumab	Monoclonal antibody	NCT03067844	294 patients with acute myocardial infarction undergoing percutaneous coronary intervention and receiving statin therapy	To determine the effect of alirocumab on plaque volume after 52 weeks	September 2021
		NCT03207945	140 individuals with HIV with known CVD or risk factors	To determine whether alirocumab can improve arterial inflammation and endothelial function in the setting of HIV infection	November 2021
Evolocumab	Monoclonal antibody	NCT02624869	163 subjects ages 10–17 with homozygous or heterozygous familial hypercholesterolemia	To evaluate safety and efficacy of evolocumab in pediatric subjects	June 2021
		NCT02867813	5037 participants with clinically evidence atherosclerotic CVD on statin therapy who completed the FOURIER study	To assess the long-term safety of evolocumab in subjects who completed the FOURIER study	December 2021
IBI306	Monoclonal antibody	NCT04031742	30 Chinese participants with homozygous familial hypercholesterolemia	To evaluate safety and efficacy of IBI306	January 2021
		NCT04759534	148 Chinese participants with heterozygous familial hypercholesterolemia	To evaluate safety and efficacy of IBI306	June 2021
Inclisiran sodium	Small interfering RNA	NCT03705234	15000 participants with CVD	To determine if inclisiran lowers the risk of heart attacks and strokes	December 2024
Lerodalcibep (LIB003)	Recombinant fusion protein of PCSK9-binding domain and human serum albumin	NCT04034485	70 patients with homozygous familial hypercholesterolemia receiving lipid-lowering therapy	To compare safety, tolerability, and serum LDL-C levels of LIB003 and evolocumab	May 2022

Note: HIV—Human immunodeficiency virus, CVD—Cardiovascular disease, RNA—Ribonucleic acid, PCSK9—proprotein convertase subtilisin/kexin type 9.
